# Adaptive bulk motion exclusion for improved robustness of abdominal magnetic resonance imaging

**DOI:** 10.1002/nbm.3830

**Published:** 2017-09-08

**Authors:** Bjorn Stemkens, Thomas Benkert, Hersh Chandarana, Mark E. Bittman, Cornelis A.T. Van den Berg, Jan J.W. Lagendijk, Daniel K. Sodickson, Rob H.N. Tijssen, Kai Tobias Block

**Affiliations:** ^1^ Department of Radiotherapy University Medical Center Utrecht the Netherlands; ^2^ Center for Advanced Imaging Innovation and Research (CAI^2^R), Department of Radiology New York University School of Medicine New York NY USA; ^3^ Bernard and Irene Schwartz Center for Biomedical Imaging, Department of Radiology New York University School of Medicine New York NY USA

**Keywords:** abdominal imaging, bulk motion, motion correction, prospective motion detection, real‐time motion detection

## Abstract

Non‐Cartesian magnetic resonance imaging (MRI) sequences have shown great promise for abdominal examination during free breathing, but break down in the presence of bulk patient motion (i.e. voluntary or involuntary patient movement resulting in translation, rotation or elastic deformations of the body). This work describes a data‐consistency‐driven image stabilization technique that detects and excludes bulk movements during data acquisition. Bulk motion is identified from changes in the signal intensity distribution across different elements of a multi‐channel receive coil array. A short free induction decay signal is acquired after excitation and used as a measure to determine alterations in the load distribution. The technique has been implemented on a clinical MR scanner and evaluated in the abdomen. Six volunteers were scanned and two radiologists scored the reconstructions. To show the applicability to other body areas, additional neck and knee images were acquired. Data corrupted by bulk motion were successfully detected and excluded from image reconstruction. An overall increase in image sharpness and reduction of streaking and shine‐through artifacts were seen in the volunteer study, as well as in the neck and knee scans. The proposed technique enables automatic real‐time detection and exclusion of bulk motion during MR examinations without user interaction. It may help to improve the reliability of pediatric MRI examinations without the use of sedation.

**Abbreviations used**
AF: artifacts
CC: correlation coefficient
CHESS: chemical shift selective
FFT: fast Fourier transformation
FID: free induction decay
FOV: field of view
HIPAA: Health Insurance Portability and Accountability Act
ICE: image calculation environment
IRB: Institutional Review Board
IQ: image quality
LE: liver edge sharpness and hepatic vessel clarity
MR: magnetic resonance
MRI: magnetic resonance imaging
RF: radiofrequency
SAD: sum of absolute differences
3D: three‐dimensional

## 1 INTRODUCTION

Motion poses a key challenge for diagnostic three‐dimensional (3D) magnetic resonance (MR) examination of the abdomen. Both physiological motion, such as respiratory and cardiac motion, and bulk motion, caused by bulk body movements or coughing, can impair the image quality and result in non‐diagnostic images.^1^, ^2^, ^3^ Various strategies have been proposed to handle physiological motion‐related artifacts. Common approaches to avoid respiratory motion artifacts include breath‐hold^4^ and respiration‐gated acquisitions.^5^, ^6^ However, many patients are unable to suspend respiration long enough to acquire sufficient data (often >15 s). Moreover, pediatric patients may not be able to hold their breath at all, and they can perform sudden movements. Therefore, general anesthesia or deep sedation is often necessary to obtain diagnostic images in these patients.^7^ The disadvantages of sedation include health risks,^8^ as well as increased logistical complexity and costs. As an alternative, gated or triggered acquisitions can be employed, using either respiratory bellows or navigators, which result in significantly reduced scan efficiency. Furthermore, respiratory bellows cannot differentiate between respiratory‐ and non‐respiratory‐related motion. Navigators, on the other hand, may affect the contrast of the imaging volume or interrupt the steady state. A comprehensive review of motion compensation techniques can be found in Zaitsev et al.,^2^ van Heeswijk et al.^3^ and MacLaren et al.^9^

In recent years, free breathing approaches based on non‐Cartesian *k*‐space acquisition have become popular.^10^, ^11^, ^12^, ^13^ Residual respiration artifacts can additionally be removed by weighting the data with the amount of motion,^14^, ^15^ or by reconstructing multiple respiratory phases.^16^ However, these techniques cannot correct for bulk motion, which includes voluntary and involuntary patient movement resulting in translation, rotation or elastic deformation of the body.^3^

One possibility to remove bulk motion is to exclude corrupted data retrospectively.^17^ Because the duration and number of intervals with bulk motion is not known in advance, *k*‐space must be acquired with significant oversampling to provide sufficient *k*‐space coverage. This prolongs the scan time unnecessarily if no (or only short) intervals of bulk motion occur, or results in residual undersampling artifacts if more bulk motion than expected occurs.

Prospective methods^18^, ^19^ either reacquire motion‐corrupted data^18^ or stop the acquisition when patient motion occurs,^19^ based on motion information derived from navigator scans. Intermediate navigator acquisition, however, prolongs the scan time and may not be compatible with all sequence types.

The aim of this work was to develop a novel, time‐efficient, adaptive, data‐consistency‐driven image stabilization technique to detect and exclude bulk movements. This was achieved by calculating a consistency measure for the acquired data in real time and continuing the acquisition until sufficient consistent data had been acquired. Without user interaction, the technique automatically detects motion‐free data windows and stops the scan if a window with sufficient length has been identified. The method was evaluated in six healthy volunteers.

Although the proposed technique efficiently rejects data corrupted by bulk motion, it intentionally does not compensate for respiratory motion, which remains visible in the motion‐stabilized images. Residual respiratory motion can be removed in a second processing step by applying the previously described XD‐GRASP approach^16^ to the motion‐stabilized data, which is demonstrated for a free breathing abdominal scan. To show that the technique can be applied for other applications, additional head/neck and knee examinations were performed.

## 2 METHODS

### 2.1 Overall strategy

The strategy of the proposed technique is to obtain a consistent data window that is not corrupted by bulk motion and that contains sufficient samples to reconstruct images with diagnostic quality. To this end, *k*‐space samples are acquired continuously and a real‐time mechanism evaluates whether or not bulk motion has occurred.

Several approaches exist to extract motion from the acquired data, commonly referred to as self‐navigation techniques. One option is to detect motion from non‐phase‐encoded *k*‐space center lines.^16^, ^20^ Although this can be accomplished efficiently for stack‐of‐stars sequences^16^ and other non‐Cartesian 3D trajectories, for Cartesian 3D sampling it requires the acquisition of additional non‐phase‐encoded *k*‐space lines, which prolongs the scan time.^20^ Alternatively, free induction decay (FID) navigators can be acquired, which sample the center of *k*‐space without spatial encoding immediately after the radiofrequency (RF) excitation.^21^, ^22^, ^23^, ^24^ This has only slight impact on TE, requires no additional RF pulses and can be applied to many different *k*‐space trajectories. The FID approach is used in this work to derive a motion signal. It has been shown previously that variations in the signal intensity ratio between different receive coils allow the identification of bulk motion.^20^

**Figure 1:**
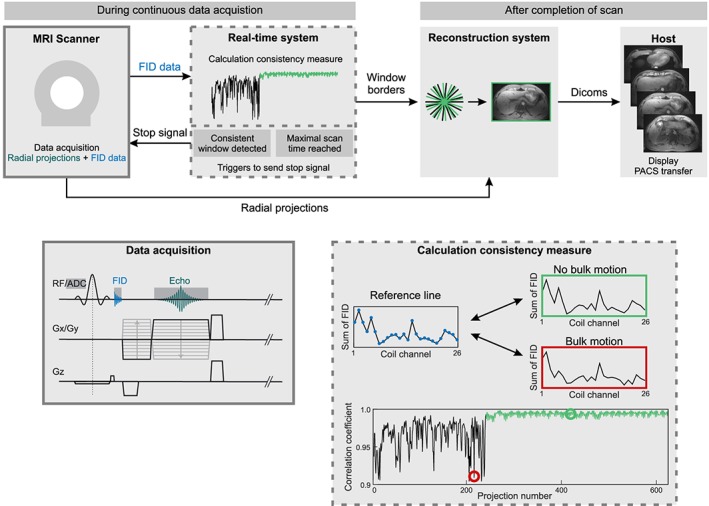
Schematic overview of the proposed bulk motion exclusion technique. A radial stack‐of‐stars three‐dimensional (3D) sequence has been modified to acquire a free induction decay (FID) signal after radiofrequency (RF) excitation. The FID samples are transferred to the real‐time processing system, where the consistency of the current projection with a reference projection is calculated based on the correlation coefficient. When sufficient motion‐free data are available or if the maximum scan time has been reached, a stop signal is sent to the scanner and images are reconstructed from the accepted data window

When bulk motion has been detected, data samples are rejected until the subject holds still. The scan is stopped automatically when a predefined window of non‐corrupted consecutive *k*‐space lines has been acquired. Data samples affected by bulk motion are discarded in the current implementation because it cannot be assumed that the absolute positions of the patient before and after bulk motion are identical. If no such data window can be found within a user‐defined maximal scan time (e.g. when the subject moves continuously), the best available window (i.e. longest window without bulk motion) is identified and used for image reconstruction. The entire procedure is schematically shown in Figure 1. It is important to note that, if the subject does not move during the examination, the technique does not prolong the scan time relative to a non‐stabilized examination.

### 2.2 Implementation

Data are acquired using a *T*_1_‐weighted radial 3D gradient‐echo sequence with both RF and gradient spoiling.^10^ The stack‐of‐stars scheme with Cartesian encoding along the *k*_*z*_ dimension and radial sampling in the *k*_*x*_–*k*_*y*_ plane is used for volumetric *k*‐space coverage. After spectral fat suppression using a chemical shift selective (CHESS) pulse, all partitions for one radial angle are acquired with centric reordering before the readout scheme is repeated for subsequent radial projections. Golden‐angle ordering with an angular increment of 111.246°^25^ is used for continuous data acquisition. To obtain motion information, 32 samples of the FID signal are acquired after rewinding the slice selection gradient, which increases the TE by less than 0.5 ms. The entire data acquisition scheme is shown in Figure 1.

The FID signals are transferred to the real‐time control unit of the image calculation environment (ICE) framework where the detection of bulk motion has been implemented. To reduce the processing latency, only the FID signals from the center *k*‐space partition are sent. The first and last four samples of each FID signal are discarded to account for pre‐ and post‐ringing. The remaining samples are summed. For each projection, the summed values from the different coils are concatenated into a projection vector of length *N*_coil_.

Motion is detected by calculating the correlation coefficient between a reference projection vector and all other projection vectors.^20^ The correlation coefficient CC is defined as:

$${\mathrm{CC}}_{x,y}=\frac{\sum_{i=1}^{N_{\text{coil}}}\left[\left(x_{i}-\bar{x}\right)\left(y_{i}-\bar{y}\right)\right]}{\sqrt{\frac{1}{N_{\text{coil}}-1}\sum_{i=1}^{N_{\text{coil}}}{\left(x_{i}-\bar{x}\right)}^{2}}\sqrt{\frac{1}{N_{\text{coil}}-1}\sum_{i=1}^{N_{\text{coil}}}{\left(y_{i}-\bar{y}\right)}^{2}}}$$

where *x*_*i*_ and *y*_*i*_ denote the *i*th entry of the current projection vector *x* and reference projection *y*. $\bar{x}$ and $\bar{y}$ are the mean values of both vectors.

A low correlation coefficient implies that the load distribution of the coil elements has changed. This indicates that the patient position has been altered between the projections. As it is not known beforehand if and when bulk motion occurs, the reference projection vector, which is defined as the projection with highest overall correlation to all previously acquired projections, is dynamically updated after each projection. The calculation of all correlation coefficients is computationally demanding and cannot always be performed in real time. In the current implementation, the reference projection is therefore only calculated within a sliding window containing the previous 100 projections. Outlier projections, which indicate bulk motion, are identified using a user‐defined acceptance threshold for the correlation coefficient.

### 2.3 Imaging studies

All volunteer scans were approved by the Institutional Review Board (IRB) and were Health Insurance Portability and Accountability Act (HIPAA) compliant. Written informed consent was obtained for all imaging studies.

#### 2.3.1 Abdominal imaging

Six healthy volunteers (three men, three women; age, 30.3 ± 3.3 years) were scanned on a 3‐T scanner (MAGNETOM Skyra, Siemens Healthineers, Erlangen Germany) equipped with a 24‐element spine coil array in combination with an 18‐element body coil array. For each volunteer, four separate free breathing scans were performed during which the subjects were asked to perform different motion tasks (Table 1). In addition, a scan without bulk motion was performed as reference. Data were acquired with the described 3D radial stack‐of‐stars sequence. The maximum scan time was set to 5 min 11 s, which corresponds to a total of 1500 projections. The length of the acceptance window was set to 400 projections (1 min 23 s) to fulfill the Nyquist criterion for radial sampling. A threshold of 0.975 was used to differentiate between regular breathing motion and bulk motion. This value was chosen empirically after the evaluation of results with different thresholds (an example is shown in Figure S1, see Supporting Information). For all scans, the liver was covered with 72 axial slices using the following sequence parameters: field of view (FOV), 350 × 350 × 216 mm^3^; slice resolution, 72%; flip angle, 12°; TR/TE = 3.35/1.71 ms; readout bandwidth, 890 Hz/pixel. Using a matrix size of 256 × 256 pixels, the resulting resolution was 1.37 × 1.37 × 3.0 mm^3^.

**Table 1:** Instructions provided to the volunteers for the four different scans. The scan was stopped automatically when a user‐defined number of consistent projections had been acquired

Motion task
Free breathing (~45 s)–bulk motion (~30 s)–free breathing (until scan stops)
Bulk motion (~30 s)–free breathing (until scan stops)
Free breathing (~45 s)–change in position–free breathing (until scan stops)
Random motion (~45 s)–free breathing (until scan stops)

#### 2.3.2 Neck and knee imaging

To demonstrate the applicability of the technique to other body areas, both a neck scan and a knee scan were performed in one volunteer each. For the neck scan, the subject was asked to perform random head motion during the scan. A 3‐T scanner (MAGNETOM Skyra, Siemens Healthineers) equipped with a 20‐element combined head/neck coil array was used to acquire data with the radial stack‐of‐stars sequence. The length of the acceptance window was set to 400 projections (1 min 47 s) and the maximum scan time was set to 6 min 42 s (1500 projections). Seventy‐two axial slices with the following imaging parameters were used to cover the neck area: FOV, 200 × 200 × 216 mm^3^; matrix size, 256 × 256 pixels; resolution, 0.78 × 0.78 × 3 mm^3^; slice resolution, 72%; flip angle, 12°; TR/TE = 4.40/2.25 ms; readout bandwidth, 650 Hz/pixel.

For the knee scan, the subject was instructed not to move during the first 40 s of the scan, and then to move the knee for 50 s, and to suspend motion for the remainder of the scan. A radial stack‐of‐stars dataset was acquired using a 3‐T scanner (MAGNETOM Prisma, Siemens Healthineers) equipped with a 15‐element knee coil array (QED, Cleveland, OH, USA). The following imaging parameters were used to cover the knee with 72 axial slices: FOV, 190 × 190 × 144 mm^3^; matrix size, 256 × 256 pixels; resolution, 0.74 × 0.74 × 2 mm^3^; slice resolution, 72%; flip angle, 12°; TR/TE = 4.51/2.30 ms; readout bandwidth, 670 Hz/pixel. The maximum scan time was set to 6 min 48 s (1500 projections) and the length of the acceptance window was set to 400 projections (1 min 49 s). Because breathing motion does not occur in a knee examination and because bulk movements are expected to be less severe, the correlation values are expected to be higher than for abdominal scans. Therefore, the threshold value was increased to 0.995.

### 2.4 Image reconstruction

After real‐time detection of the motion‐free data window, the window borders are transferred to the reconstruction pipeline. Final images are reconstructed using only the projections from within this data window. Image reconstruction is performed using custom‐developed modules in the ICE framework. First, phase encoding along the slice direction is removed by applying an inverse fast Fourier transformation (FFT). After density compensation with a Ram–Lak filter, images are reconstructed with a non‐uniform FFT, which uses a Kaiser–Bessel window as interpolation kernel for the gridding step.^26^ Because the processing has been implemented into the reconstruction framework of the scanner, built‐in standard post‐processing steps, such as image distortion correction, are performed automatically. Additional specific corrections, such as gradient delay correction, have not been implemented. The sum‐of‐squares approach is used to combine signals from different coil channels. Inhomogeneities in the RF receive profiles are compensated for by enabling ‘prescan normalize’, which is an image homogeneity filter. Calculated correlation coefficients of the bulk motion detection and the borders of the accepted window are written into a text file, which is exported for optional visualization of the motion curves.

To demonstrate the performance of the proposed image stabilization technique, the automatically reconstructed images are compared with two additional reconstructions using: (1) the same window length but starting from the first projection; and (2) all acquired projections, corresponding to the same maximum scan duration.

To show the compatibility of the proposed method with additional respiratory motion compensation, the XD‐GRASP approach^16^ was applied to the accepted data from one volunteer dataset. The FID navigator data allow not only the detection of bulk motion, but also of respiratory motion. For this application, an additional low‐pass filter was applied to the motion signal. The information was then used to sort the data into four respiratory states, ranging from end‐expiration to end‐inspiration. Each frame then consisted of 100 projections, which would result in visible undersampling artifacts with standard gridding. Therefore, iterative reconstruction was performed which minimizes the total variation along the respiratory dimension to suppress radial undersampling artifacts. As a final step, the respiratory phases were registered using a non‐rigid 3D optical flow algorithm^27^ and averaged to generate one final high‐quality image.

### 2.5 Validation

To validate the correlation measure for the detection of bulk motion, four abdominal scans of one volunteer were recorded with a video camera (PowerShot SX170IS, Canon Inc., Tokyo, Japan), which was mounted outside the scanner bore. The start of the MR acquisition was determined using the audio signal. Bulk motion was detected in the videos by calculating the sum of absolute differences (SAD) between consecutive frames. When no motion or only slight motion occurs between frames, such as regular breathing, the SAD value is very small. Bulk motion, in contrast, results in a high SAD value. For each scan, the SAD values were plotted over time and visually compared with the corresponding plot of the correlation coefficients.

### 2.6 Reader evaluation

Results from the abdominal scans of the six volunteers were rated by two independent and blinded board‐certified radiologists (H.C. and M.E.B.) with 9 and 5 years of experience, respectively. Subject name and acquisition parameters were stripped from all images. Before reviewing the datasets in randomized order, both readers were briefed using sample images to calibrate the reading. The categories image quality (IQ), liver edge sharpness and hepatic vessel clarity (LE) and artifacts (AF) were scored on a five‐point Likert‐type scale (1, poor; 5, excellent). A free breathing scan without bulk motion was used as reference and reconstructed with conventional gridding. For all scans with motion tasks, images reconstructed from the automatically selected window, images using the same window length but starting at the first projection, and images using all acquired data were scored. Results were averaged over both readers and the mean, median and standard deviation were calculated.

## 3 RESULTS

### 3.1 Validation

**Figure 2:**
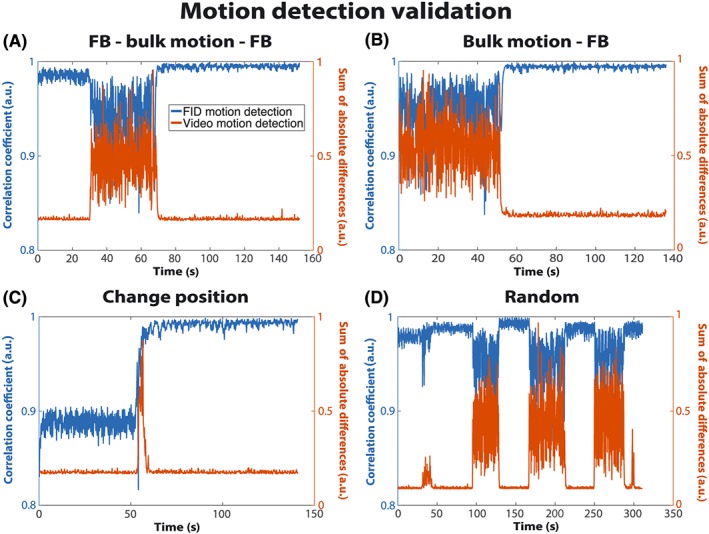
Correlation coefficients calculated from the free induction decay (FID) navigator (blue) and the sum of absolute differences (SAD) from the video (orange) for the four scans of one volunteer. When bulk motion occurred, the correlation coefficient decreased, whereas SAD increased. As a result of noise and respiratory motion, the SAD values are non‐zero even when no bulk motion occurred. FB, free breathing

Figure 2 summarizes the results from the video validation for one of the volunteers. For all scans, the motion curve detected with the proposed technique (blue) shows high agreement with the motion detected on video (orange). When bulk motion occurs, the correlation coefficient decreases, whereas SAD increases because of the larger differences between the video frames (Video S1, see Supporting Information). Because of noise and respiration, SAD values ranged between 0.1 and 0.2 even in intervals without bulk motion. Figure 2c shows plots from the scan when the volunteer changed position. The two positions are clearly distinguishable from the FID motion curve, whereas the video‐based detection only indicates the moment when the subject moved. Figure 2d shows different periods of free breathing and bulk motion. The initial peaks around 40 s were caused by coughing. The last peak around 300 s was caused by head motion, which was not identified by the FID navigators because the RF pulse did not excite this part of the body. Because a window of 400 consistent projections was not detected, the scan was stopped after 311 s and the best window, between 50 and 100 s, was used for image reconstruction.

### 3.2 3D abdominal imaging with bulk motion

**Figure 3:**
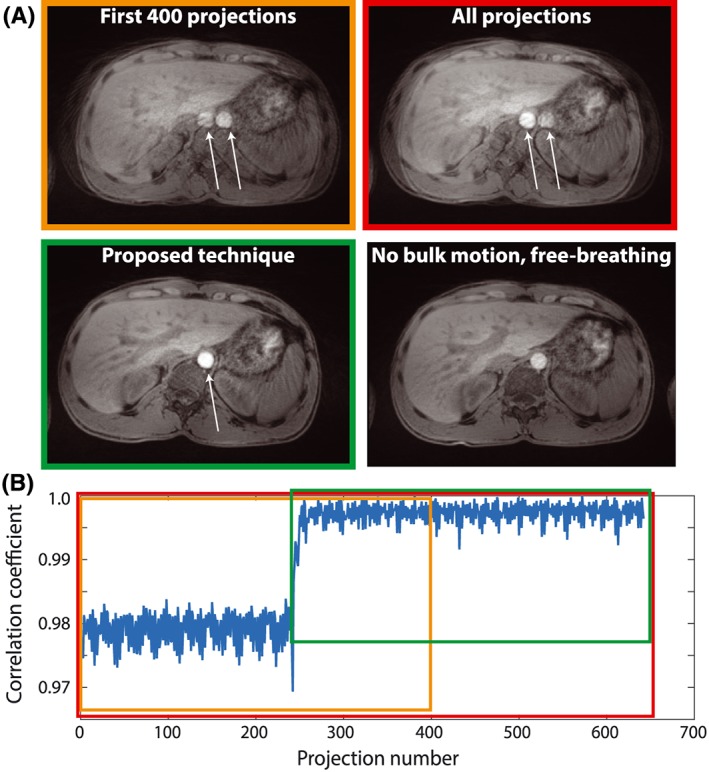
Results from a volunteer scan with position change. (a) reconstructions from the first 400 projections (orange), all projections (red), accepted projections (green) and a free breathing reference scan without bulk motion. White arrows indicate shine‐through artifacts that are reduced using the proposed technique. (b) correlation coefficient and data windows from (a)

Figure 3 compares images reconstructed from the first 400 projections (orange), all acquired projections (red) and only the accepted projections (green) with the reference free breathing acquisition without bulk motion for a volunteer who changed position. In addition, the time course of the correlation coefficient is shown (Figure 3b). A major reduction in shine‐through artifacts can be seen with the proposed technique. Although the reconstruction using all projections shows fewer streaking artifacts and higher SNR, the images are non‐diagnostic because of the two overlapping positions of the subject. These artifacts are removed with the proposed technique, which provides image quality comparable with the reference free breathing acquisition. The slightly hyperintense area in the center of the images is caused by overcorrection of the RF receive profile by the vendor‐provided ‘prescan normalize’ algorithm.

**Figure 4:**
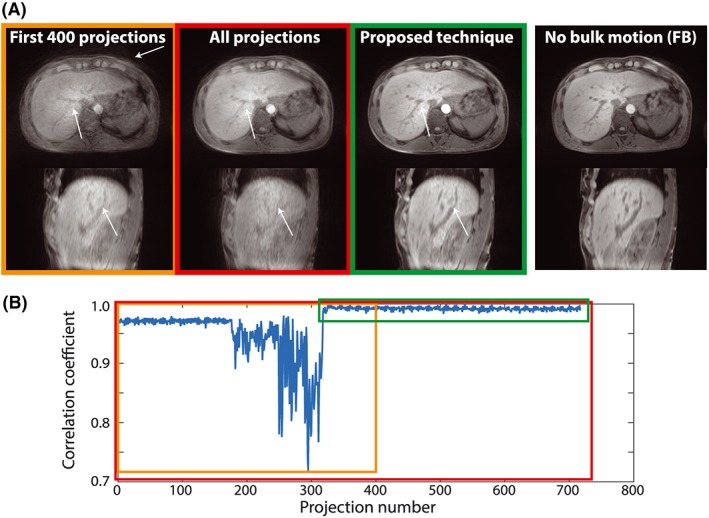
Results from a volunteer scan starting with free breathing, followed by bulk motion, followed by free breathing. (a) reconstructions in axial and sagittal orientations; (b) data windows for the initial 400 projections (orange), all projections (red) and accepted projections (green). White arrows indicate areas of increased/decreased artifacts. The proposed technique does not correct for respiratory motion, resulting in residual blurring at the diaphragm comparable with the free breathing reference scan (right). FB, free breathing

Figure 4 shows the results from another subject who started to move after 36 s (175 projections), followed by normal free breathing after 65 s (317 projections). Images from the initial 400 projections show substantial motion artifacts in both the axial and sagittal views. The artifact strength is slightly reduced in the axial view when all 717 projections are used, but is worse in the sagittal view. Best image quality is achieved if only the accepted projections are used, with similar image quality as the reference reconstruction in axial orientation. Respiratory motion remains visible, resulting in blurring at the top of the diaphragm in the sagittal view, which appears slightly lower in the reference acquisition without bulk motion.

### 3.3 Reader evaluation

Scores from the reader evaluation are shown in Table 2. For all motion tasks, the proposed technique (‘Stabilized’) achieved the highest scores in all three rated categories (IQ, LE and AF). When scores were averaged over the different tasks and over both readers, image quality with the proposed technique was 3.92 ± 0.37, in contrast with 2.63 ± 1.07 when using all projections and 1.83 ± 0.49 when using the initial data window. The image quality score for the reference scan was 4.33 ± 0.88.

**Table 2:** Scores from the volunteer study on a five‐point Likert‐type scale (1, poor; 5, excellent), averaged over two readers. Results are shown for all performed scans, including the reference scan and four motion tasks. Each image was rated in the categories IQ (image quality), LE (liver edge sharpness and hepatic vessel clarity) and AF (artifacts)

		IQ		LE		AF	
		Mean (SD)	Median (range)	Mean (SD)	Median (range)	Mean (SD)	Median (range)
FB (reference)	–	4.33 (0.88)	4.75 (3–5)	4.22 (0.88)	4.75 (3–5)	**4.00** (1.10)	4.5 (2–5)
FB–bulk–FB	Stabilized	4.08 (0.66)	4.5 (3–4.5)	4.17 (0.75)	4.5 (3–5)	3.83 (0.82)	4 (2.5–4.5)
	Initial	1.67 (0.52)	2 (1–2)	1.75 (0.61)	2 (1–2.5)	1.67 (0.52)	2 (1–2)
	All	2.42 (0.80)	2.75 (1–3)	2.42 (0.74)	2.5 (1–3)	2.33 (0.75)	2.5 (1–3)
Bulk–FB	Stabilized	**4.33** (0.68)	4.5 (3–5)	**4.42** (0.49)	4.5 (3.5–5)	3.75 (0.69)	4 (3.5–4.5)
	Initial	2.50 (1.00)	2.5 (1.5–4)	2.50 (1.00)	2.5 (1.5–4)	2.42 (0.86)	2.25 (1.5–4)
	All	3.75 (1.04)	3.75 (2–5)	3.67 (0.98)	3.75 (2–5)	3.42 (1.02)	3.5 (1.5–4.5)
FB–position change–FB	Stabilized	3.50 (1.00)	3.5 (2–5)	3.42 (1.24)	3.25 (1.5–5)	3.50 (0.84)	3.5 (2–4.5)
	Initial	1.33 (0.41)	1.25 (1–2)	1.83 (0.61)	1.75 (1–2.5)	1.17 (0.26)	1 (1–1.5)
	All	1.25 (0.42)	1 (1–2)	1.58 (0.58)	1.5 (1–2.5)	1.33 (0.61)	1 (1–2.5)
Random–FB	Stabilized	3.75 (1.13)	4 (2–5)	3.92 (1.07)	4.25 (2–5)	3.50 (0.89)	3.75 (2–4.5)
	Initial	1.83 (0.82)	1.75 (1–3)	1.75 (0.82)	1.5 (1–3)	1.75 (0.52)	1.75 (1–2.5)
	All	3.08 (0.74)	3.25 (2–4)	3.33 (0.52)	3.5 (2.5–4)	3.00 (0.63)	3.25 (2–3.5)

FB, free breathing; SD, standard deviation.

Bold values state the highest scores for the three categories.

When the different motion tasks were treated separately, the bulk–free breathing scans achieved the highest image quality score (4.33 ± 0.68), which was identical to the reference scan. This was followed by free breathing–bulk–free breathing (4.08 ± 0.66), random (3.75 ± 1.13) and change position (3.50 ± 1.00). Unlike the non‐stabilized scans, the proposed technique achieved diagnostic image quality (score > 3) for all motion tasks.

### 3.4 Motion‐resolved 3D abdominal imaging

**Figure 5:**
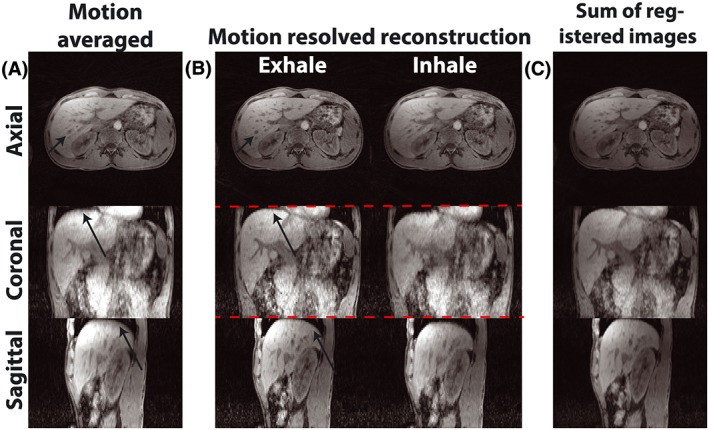
Respiration‐resolved reconstruction. (a) motion‐averaged reconstruction from bulk motion‐stabilized data with blurring caused by respiration. (b) motion‐resolved images for in‐ and exhale phases (note the varying liver position relative to the red line), showing increased sharpness but residual undersampling. (c) averaged image after registration to the fixed respiratory phase. Black arrows indicate areas of increased/decreased respiratory blurring

Figure 5 shows images with and without additional respiratory motion compensation. In the motion‐averaged image (Figure 5a), blurring can be seen at the tip of the liver in the coronal and sagittal views, as well as around the liver vessels on the axial view (see black arrows). The individual respiratory phases from the XD‐GRASP reconstruction show much sharper edges and contours in all views and phases (Figure 5b). Residual undersampling artifacts are visible, which are incompletely removed by the motion‐resolved reconstruction. After averaging the non‐rigidly registered volumes, these artifacts are largely removed. The respiratory motion signal extracted from the FID signal correlated well with the self‐navigation signal extracted from the center of *k*‐space (not shown).^16^

### 3.5 3D head/neck and knee imaging

**Figure 6:**
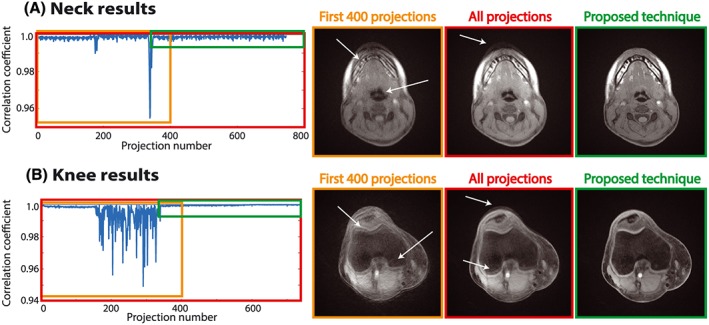
Results from neck (a) and knee (b) scans. Reconstructions from accepted data windows without bulk motion (green) compared with non‐stabilized reconstructions (red, orange). Blurring and streaking (white arrows) are decreased and the overall image quality is improved

Figure 6a shows results from the head/neck scans during which the volunteer moved the head twice. The rotations are clearly noticeable in the correlation plot around projection numbers 180 and 330. Although the movements and corresponding artifacts are more subtle than for abdominal bulk motion, reduced artifacts are obtained with the proposed technique, indicated by the white arrows.

Results from the knee scan are shown in Figure 6b. The correlation plot clearly reveals motion between projections 150 and 340. When these data are included in the reconstruction, image quality is severely degraded and artifacts become visible, as indicated by the white arrows. The proposed technique identified a motion‐free interval, resulting in improved image quality without visible motion artifacts.

## 4 DISCUSSION

Although a variety of techniques for motion‐robust abdominal imaging have been proposed in the literature, the occurrence of bulk motion still poses an unresolved problem and can result in non‐diagnostic image quality or misinterpretation of images. This work describes a new approach to detect and exclude non‐respiratory bulk motion from continuously acquired 3D scans, based on the measurement of the variations in the signal intensity distribution between different elements of a multi‐channel receive coil. Using recordings of volunteer scans, it has been validated that the variations in FID intensity ratios correlate with bulk motion (see Video S1). Six volunteers were scanned and asked to perform different types of motion, including free breathing, bulk motion, position changes, coughing, moving the coil or arms and rolling around. For all motion types, the proposed technique was able to detect motion‐free data windows. In the abdominal reader study, images reconstructed without the proposed technique were mostly non‐diagnostic and achieved scores well below three. When the stabilization approach was applied, diagnostic image quality (score > 3) was obtained for all motion tasks and categories. Compared with the free breathing reference scan, the bulk–free breathing task achieved identical image quality. Scores were slightly lower for the position change and random motion task, which can be explained by the finding that subtle bulk motion was misinterpreted as breathing motion and therefore not excluded. This behavior can be improved by a more thorough optimization of the acceptance threshold or by using patient‐specific calibration of the threshold value. An additional reason for the slight discrepancy between the reference scan and the stabilized scan might be that the ‘prescan normalize’ intensity correction uses calibration data from a different patient position. When the subject changes position, the ‘prescan normalize’ correction profile mismatches the new position, which can result in spurious intensity modulation and may affect the image quality score.

The technique has several advantages over previously described methods for retrospective bulk motion correction.^17^, ^28^ First, it is fully automatic and does not require user interaction. As a minimum number of projections is always acquired, images can be reconstructed directly on the scanner without the need for complex processing, such as compressed sensing^29^ or registration algorithms.^28^ Second, the technique is time efficient, as no assumptions are made with regard to if, when and how much bulk motion might occur during the scan. This is important because some patients may start to move when the scan starts (because of acoustic noise onset), whereas others may move at a later time (because of discomfort). If no bulk motion occurs, only the minimum scan time is used, which may not be the case for retrospective bulk motion removal.^17^ Third, to ensure that the scan time is not unreasonably prolonged in the presence of ongoing bulk motion, a maximum allowed scan time can be defined. The longest bulk motion‐free window is then used for reconstruction. If the number of projections in this window is smaller than the required minimum number of projections, the threshold is slightly reduced until a window with sufficient data is found. This mechanism was implemented because images with too few projections would be affected by severe undersampling artifacts. Lastly, another advantage of the proposed technique is that it does not reacquire data,^18^ but continues the golden‐angle acquisition until sufficient consecutive motion‐free data have been recorded. This results in higher scan efficiency than the reacquisition of the data.

In the current implementation, only consecutively acquired profiles are accepted for reconstruction. This restriction could be relaxed by using all data except for motion‐corrupted profiles, which would result in improved time efficiency. However, the body position of the patient before and after bulk motion cannot be assumed to be identical. This holds true especially for abdominopelvic applications (as shown in Figures 2 and 3), but even for somewhat mild bulk motion, such as coughing. Therefore, reconstruction from non‐consecutive data would increase blurring and result in an overlay of different patient positions, which has previously been reported for body^17^ and head^28^ imaging. Nonetheless, for minor movements in relatively rigid body areas, such as swallowing motion in the neck, only the projections affected by bulk motion could be removed to improve scan efficiency. Furthermore, if the motion pattern contains multiple discrete states, it would be possible to identify the motion‐free states and treat the corresponding *k*‐space data as an additional reconstruction dimension, in a similar manner to XD‐GRASP. If there is sufficient correlation between the states, such motion‐extended reconstruction could provide additional scan acceleration and/or SNR improvement. However, the achievable improvement would be highly variable and dependent on the motion type and strength that occurred during each individual scan.

The threshold value for acceptance was chosen heuristically, based on retrospective processing of several *in vivo* datasets. For the abdominal and neck scans, a value of 0.975 was found to be a reasonable tradeoff between the exclusion of bulk motion and acceptance of respiratory motion for all volunteers. As shown in Figure S1, a threshold value between 0.950 and 0.980 gives comparable image quality, suggesting that the method is not very sensitive to the exact value of the threshold parameter. It is important to note, however, that the threshold value can depend on the application and type of motion. For example, a higher threshold was required for knee examination. Thus, it might be necessary to select a fixed threshold value for each distinct application. In addition, the threshold value may depend on the receive coil setup and field strength. One strategy to address this dependence could be to implement an automatic calibration mechanism that retrospectively estimates the optimal value using image‐based artifact measures until a stable value has been identified. This value could then be reapplied to scans with the same coil and sequence setup.

For several of the abdominal acquisitions, the number of accepted projections was slightly higher than the required 400 projections. This can be explained by the dynamic update mechanism of the reference projection and correlation coefficient. In the moment when the reference projection is updated, the correlation coefficient is recalculated and it is possible that more than 400 projections have already been acquired that are consistent with the new reference projection. Although resulting in only minor reduction of the method's efficiency, this behavior can potentially be improved by increasing the sliding window used to calculate the reference projection or by averaging consecutive accepted projections into a mean reference projection.

The proposed method is versatile and generalizable. In this initial implementation, a golden‐angle radial stack‐of‐stars *T*_1_‐weighted 3D gradient‐echo sequence was used. Other continuous sampling techniques could also be employed, such as variable density sampling and radial view ordering,^15^ Golden‐step Cartesian,^30^ blipped multi‐echo 3D radial stack‐of‐stars^31^ or 3D radial.^32^ Some of these trajectories have inherent self‐navigation capabilities. As a result of the acquisition of the FID navigator, the proposed method can also be applied to Cartesian sampling strategies.^21^, ^33^ If the sampling scheme does not directly allow for continuous data acquisition, such as with conventional fast spin echo acquisition, the method could be modified to perform repeated *k*‐space acquisition until a motion‐free period has been found. The start of the reconstruction window could still be placed at the beginning of the motion‐free period in a sliding window manner. However, a limitation is that artifact‐free reconstruction is only possible if the motion‐free period is long enough for the acquisition of a full dataset. In contrast, with golden‐angle sampling, images can be reconstructed from any window length.

A further advantage of the FID navigator is that it extracts motion from the entire excited volume independent of the scan orientation, which makes it possible to acquire images in coronal, sagittal and oblique orientation.^34^ Moreover, the FID signal is not affected by gradient imperfections, such as gradient delays, whereas a self‐navigation signal extracted from the center of *k*‐space can have an additional dependence on the radial angle as a result of timing inaccuracies.^35^ Time‐dependent drifts of the FID signal, which can be a common problem with methods based on the signal phase, were also not observed in the current study, probably because the proposed technique uses the signal magnitude for calculation of the motion measure. The FID navigator can detect both bulk motion and respiration, which can be compensated for in a second step via retrospective sorting or weighting of the data, as demonstrated in the XD‐GRASP example (Figure 5). Lastly, the FID signal provides high temporal resolution as it is acquired prior to each read‐out event. Conventional self‐navigation techniques often provide lower temporal resolution, which can be insufficient for large imaging volumes or slower acquisition methods, such as radial stack‐of‐stars 3D fast spin echo acquisitions.

A limitation of the approach is that it cannot be applied to all clinical imaging protocols. For example, combination with dynamic contrast‐enhanced acquisition is not feasible, because rejection of data and scan continuation are not viable strategies when images are required at defined enhancement phases.

A further limitation is that, to date, the method has only been tested in a small number of volunteers. Evaluation in a sufficiently large patient study is necessary before reliable conclusions about the effectiveness in routine clinical application can be drawn. This will be investigated in a planned study for non‐sedated pediatric patients who often move abruptly in the magnetic resonance imaging (MRI) scanner.

## 5 CONCLUSION

This work describes a time‐efficient method for automatic detection and exclusion of bulk motion during volumetric abdominal MR examination. Bulk motion is detected by correlating FID signals across different coil elements using a real‐time implementation. The acquisition is continued until sufficient consecutive data without bulk motion have been acquired. Images from the motion‐free period are reconstructed on the scanner without additional user interaction. The technique promises to improve the robustness of abdominal MRI scans and may be of particular value for clinical examination of pediatric patients without use of sedation.

## Supporting information


**FIGURE S1** Effect of different threshold parameters. A threshold value that is too low (<0.95) results in failure to exclude bulk motion, leading to blurry images and residual artifacts. A threshold value that is too high (>0.985) results in exclusion of respiratory motion together with bulk motion, leading to short acceptance windows, increased acquisition time and/or undersampling artifacts. A reasonable trade‐off is achieved between 0.95 and 0.975

**VIDEO S1** Video showing the recording of the volunteer (top left corner), the absolute differences on a frame‐by‐frame basis (top right corner), the plot of the sum of the absolute differences (SAD, orange) and the correlation coefficient over time (blue). Only an excerpt of the video is shown to minimize its size
