# Supplementary figures and images for: Adaptive bulk motion exclusion for improved robustness of abdominal magnetic resonance imaging
Source: NMR Biomed. 2017 Sep 8;30(11):e3830. doi: 10.1002/nbm.3830 (PMC5643254; doi:10.1002/nbm.3830)

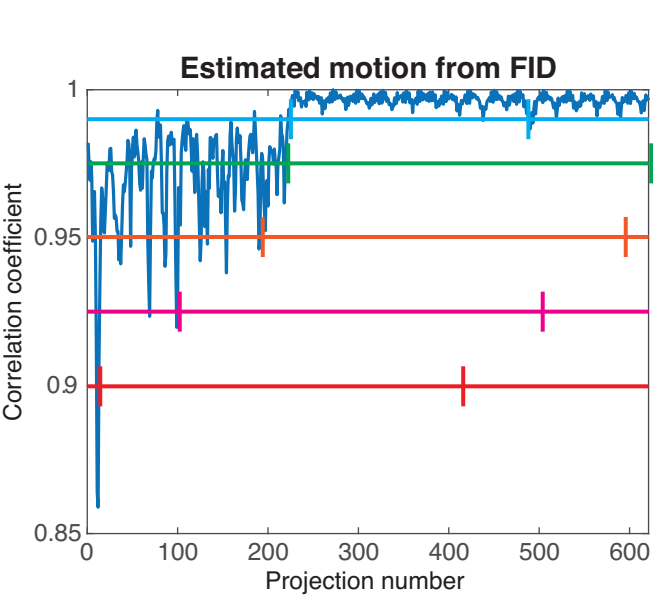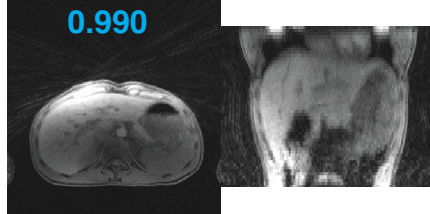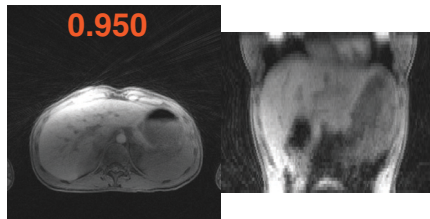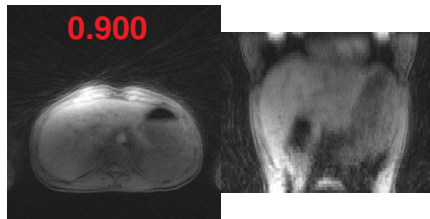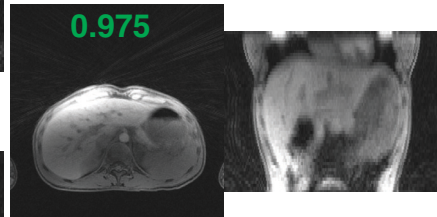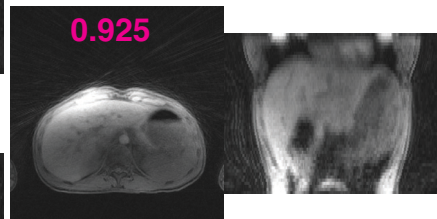

Supplement: Supplementary file 1 — FIGURE S1 Effect of different threshold parameters. A threshold value that is too low (<0.95) results in failure to exclude bulk motion, leading to blurry images and residual artifacts. A threshold value that is too high (>0.985) results in exclusion of respiratory motion together with bulk motion, leading to short acceptance windows, increased acquisition time and/or undersampling artifacts. A reasonable trade‐off is achieved between 0.95 and 0.975 [file NBM-30-na-s001.pdf]
